# Bringing Smart Home Technology to Peer-to-Peer Accommodation: Exploring the Drivers of Intention to Stay in Smart Accommodation

**DOI:** 10.1007/s10796-021-10227-4

**Published:** 2021-12-06

**Authors:** Savvas Papagiannidis, Dinara Davlembayeva

**Affiliations:** 1 Newcastle University Business School, 5 Barrack Road, Newcastle, Upon Tyne NE1 4SE UK; 2 Kent Business School, Canterbury, Kent CT27NZ UK

**Keywords:** Sharing economy, Smart homes, Smart accommodation, SMEs, Value, Hospitality, The theory of consumption values

## Abstract

COVID-19 has caused disruptions in the sharing economy for both platforms and owners, who are typically micro-businesses. Lower demand and ample supply means that users have a great deal of choice. Finding ways for properties to differentiate themselves has been a pressing need. Against this background, this paper pursued two objectives: firstly to explore the perceived functional and emotional value of smart accommodation and the factors contributing to this by adopting the Theory of Consumption Values, and secondly to examine the role of perceived value in driving intention to stay in smart accommodation in the future. 430 responses were collected to analyse the relationships among antecedents, value and intention. The results showed that the functional value of smart accommodation is associated with the perception that such accommodation represents good value for the price, smart devices are useful, they can enhance control of stay experiences, and there are resources and opportunities facilitating the use of technology. Emotional value is determined by the perception that staying in smart accommodation represents sustainable behaviour, the integration of smart home technologies offers control over the stay experience, improves the entertainment experience, aesthetics and playfulness of using technology. Emotional values are inhibited by the perception of surveillance in smart accommodation. Also, the study offers evidence of the correlation of intention with functional and emotional value. The evidence contributes to the literature by explaining the potential implications of innovative technologies for business recovery in the post-pandemic reality, exploring the applications of smart technologies in delivering tourism services, and identifying the factors in the adoption of smart homes in the hospitality sector. The findings provide practical implications for facilitating the applications of innovative technology and its adoption in home and non-home environments.

## Introduction

The Covid-19 pandemic has been a significant challenge for the sharing economy. Travel restrictions and social distancing have had a heavy impact on accommodation services rented through digital platforms (ONS, 2020); (Hossain, 2021). Following a dramatic decline in international and national travel, the accommodation sector is forecast to recover only by 50% in 2021 (OCAO, 2021). To complicate things further, the ability of the sharing economy sector to survive in the current reality has been questioned (Chen, Cheng, et al., 2020a; Hossain, 2021). Once popular due to the collaborative use of underutilised resources, the sharing business model has backfired during the pandemic (Gerwe, 2021). Travellers are reluctant to participate on such platforms, due to the health implications that the use of shared resources may have (Deloitte, 2020). In the wake of the impact of the pandemic on the accommodation industry, the profitability of such business is heavily contingent on the recovery measures undertaken by service providers (Gerwe, 2021). Given that such providers are typically small-medium sized enterprises (SMEs) with limited resources, identifying ways to differentiate their offering from others has become a pressing need. On the other hand, beyond the pandemic, new opportunities may offer added value and a competitive advantage over other accommodation types. The introduction of smart accommodation – i.e. sharing-based accommodation with integrated smart home technologies - could be such an opportunity to improve service value. The intelligence and automation capabilities of the technology make it possible to anticipate and respond to customers’ needs (Marikyan et al., 2019), which could help attract customer interest. The potential of a new service offering to minimise the negative impact of the pandemic on SMEs in the hospitality sector warrants research on users’ motivation to rent smart accommodation provided through sharing economy platforms.

Even though there is a growing stream of research focusing on the use of innovative technologies in tourism (Pai et al., 2020; Wang et al., 2020), published articles lack evidence about consumers’ intention to stay in peer-to-peer accommodation integrated with such technologies. A few papers have examined the implications of technological advances in the sharing economy, leaving the consumer perceived value of such technology in delivering enhanced services largely under-explored (Hayes et al., 2021; Rathore et al., 2020). In particular, published research has explored the potential benefits of applying blockchains, such as secure and efficient data access and processing, for online-based business models (Rathore et al., 2020). Recent developments in technology led towards the consideration of the wider applications of peer-to-peer online platforms for the energy sector, smart cities and a sustainable urban environment (Hayes et al., 2021; Obaidat & Nicopolitidis, 2016). Also, researchers adopted a provider’s perspective when examining hosts’ decision making and motivation to use smart home devices for improving peer-to-peer accommodation services (Pappas et al., 2021). Broader literature on tourism offers insights into the impact of innovative applications on travel experiences (Jeong & Shin, 2020; Li et al., 2017; Wang et al., 2020). Studies conceptually explored the role of technologies in smart tourism (Li et al., 2017), and empirically examined the effect of motivations and the characteristics of smart destination solutions on tourist experiences (Jeong & Shin, 2020; Wang et al., 2020). Despite the above evidence, still, there is limited knowledge about the user-perceived value of accommodation equipped with innovative technologies, as well as its characteristics and attributes, against which the value of services can be evaluated.

From the perspective of information systems management, the utilisation of smart home devices in travel accommodation settings represents a spillover application of technology, which is under-researched so far. Published papers have explored advanced technologies designed for home applications and utilised inside personal houses (Aldossari & Sidorova, 2020; Marikyan et al., 2021; Sequeiros et al., 2021). Evidence suggests that hedonic and utilitarian value and the perceived aspects of human interaction with smart home devices determine technology acceptance and users’ satisfaction (Aldossari & Sidorova, 2020; Sequeiros et al., 2021). The spillover application, though, entails different experiences of using the technology for extrinsic and intrinsic purposes, potentially affecting the beliefs about the features, use experiences and the outcomes of the interaction with technology (e.g. Gao & Sunyaev, 2019). In addition to the immediate benefits it can bring to the accommodation service, such a use case represents an opportunity to experiment with technologies in an actual setting. This could have implications for smart home preferences and the travellers’ interaction with the technology in their homes. Therefore, research on the factors underpinning the spillover application of technology could bring new evidence to the literature on smart home utilisation.

In view of the above, this study addresses the gap about users’ perception and motivation to rent accommodation offered through peer-to-peer platforms. Therefore, the objective of the current study is two-fold. First, the study explores the perceived value of smart accommodation and the determinants of value, which bring an understanding of the drivers of interest in a new service offering. For that purpose, we draw on the literature on tourism and smart homes to identify the factors related to (1) smart accommodation services, (2) smart home use and (3) the stay experience. These factors can differentiate smart accommodation services from traditional ones offered through peer-to-peer platforms. Subsequently, in line with the Theory of Consumption Values, the derived factors are conceptualised in relation to the functional and emotional value of smart accommodation provided through sharing economy platforms. Secondly, the study investigates users’ motivation to use such services by analysing the relationship between perceived functional and emotional value and intention to rent. To examine the proposed model, the study uses a survey design to collect data from smart home users who have experience and knowledge of smart home technology.

By addressing the above objectives, the study bridges the gap in the current body of knowledge. First, the study offers missing evidence about the customers’ perception of the value of smart accommodation. This evidence provides an understanding of the potential drivers of renting such accommodation and contributes to the research on the implications of innovative technology adoption for enhanced hospitality and tourism services (Jeong & Shin, 2020; Lee et al., 2020; Pappas et al., 2021; Wang et al., 2020). Secondly, in contrast to prior research (Aldossari & Sidorova, 2020; Hussain et al., 2009; Marikyan et al., 2021), the study extends the knowledge about the utilisation of smart home technologies in non-personal home settings. Such knowledge is timely against the backdrop of the increasing adoption of innovative technologies in the private, public and industry sectors (Budd et al., 2020; Leonardi, 2020; Papadopoulos et al., 2020). Finally, by exploring the perception of smart accommodation value by potential customers, this paper adds to the emerging literature on the applications of innovative technologies by micro entrepreneurs for transforming service propositions in the post-pandemic world (Gerwe, 2021; Pai et al., 2020; Usak et al., 2020). The findings of the study provide practical implications for facilitating the diffusion of smart home technologies and transforming accommodation services in such a way that they will create better value for customers.

The following section of the paper will provide a review of the literature on accommodation sharing and smart homes. The following hypothesis development section will present a conceptual framework and evidence supporting the relationships between the constructs. The methodology section will outline the research approach and the procedures that were used to test the model. This is followed by the results and a discussion of the findings. The paper concludes with the theoretical contributions, practical implications, limitations and future research suggestions.

## Literature Review and Theoretical Foundation

### Peer-to-Peer Accommodation

Peer-to-peer accommodation is a service enabled by online platforms, such as Airbnb, Couchsurfing**,** HomeAway, through which people rent out their underutilised rooms, flats and houses (Belk, 2014). This service has emerged as an alternative to the traditional hospitality sector, offering micro-entrepreneurial opportunities for property owners (Zhang, Bufquin, & Lu, 2019a). In accommodation sharing, platforms regulate relations between consumers (renters) and hosts, trading their personal resources and gaining economic benefits (Davlembayeva et al., 2019; Martin, 2016; Sundararajan, 2016). This form of micro-entrepreneurship can facilitate self-employment, generate income and contribute to a sustainable economic society (Davlembayeva & Papagiannidis, 2021; Zhang, Bufquin, & Lu, 2019a). Compared to other service sectors, though, the growth of such a business is more dependent on customers, due to their stronger participatory role in creating value (Nadeem et al., 2020). Hence, the literature provides a great deal of evidence about the factors driving demand in accommodation shared through online platforms.

Customer intention to participate in accommodation sharing can be categorised into three groups – values, attributes and experience (Böcker & Meelen, 2017; Guttentag et al., 2018; Lee & Kim, 2018; Li et al., 2021; Wang & Nicolau, 2017; Xu, 2020; Yu et al., 2020). Renting through platforms reflects utilitarian, social and hedonic values (Böcker & Meelen, 2017; Lee & Kim, 2018; Xu, 2020). Utilitarian value is realised when guests enjoy economic benefits, booking convenience or location accessibility (Li et al., 2021). Sharing accommodation can be economically beneficial, as it makes it possible to maximise efficiency by reducing renting costs (Tussyadiah & Pesonen, 2016; Xu, 2020). Cost reduction happens due to on-demand reuse of personal resources and the simplified transaction processing connecting providers and customers through a digital intermediary (Akbar & Tracogna, 2018; Böcker & Meelen, 2017). Social value is manifested when accommodation sharing enhances a renter’s acceptance by society and increases personal self-concept through interaction with hosts (Davlembayeva et al., 2020; Jiang et al., 2019; Johnson & Neuhofer, 2017). Hedonic value is associated with a positive experience resulting in satisfaction (Lee & Kim, 2018; Li et al., 2021).

When it comes to attributes, researchers distinguish the relative advantages of sharing-based accommodation compared to hotel services compared to independently offered properties typically run by micro-business owners (Birinci et al., 2018; Guttentag et al., 2018; Tussyadiah & Pesonen, 2016). Among the attributes considered, past research has examined, for instance, location convenience, price, diversified range of offerings and home amenities (Tussyadiah & Pesonen, 2016; Wang & Nicolau, 2017; Yu et al., 2020). Compared to hotels, the convenience of sharing-based accommodation is that it is located across residential neighbourhoods, which improves the accessibility to wider geographical areas (Guttentag et al., 2018). A relatively lower price makes it possible for guests to increase travel frequency and extend the length of stay (Tussyadiah & Pesonen, 2016). The dynamic pricing system based on the properties and the features of the accommodation enhances the customisation of services (Wang & Nicolau, 2017). This enables people to choose facilities that would best accommodate renters’ needs in relation to home specifications, pet-friendliness, check-in services and others (Yu et al., 2020). However, the lack of standards in sharing-based accommodation regarding services and facilities makes this service a subject of quality and safety concerns (Birinci et al., 2018).

As far as experience is concerned, social interaction and local authenticity were highlighted across the studies (Birinci et al., 2018; Davlembayeva et al., 2019; Guttentag et al., 2018). Sharing services entail a higher degree of interaction with hosts, although this varies depending on the type of accommodation (Guttentag et al., 2018). The involvement of hosts in the provision of additional services, such as tours around the house or the neighbourhood, was found to be important to ensure satisfaction with the stay experience (Camilleri & Neuhofer, 2017). When staying in non-tourist downtown areas and interacting with local hosts, travellers have higher engagement with the local community, culture and traditions, contributing to authentic experiences (Birinci et al., 2018).

Despite published evidence about the underpinnings of accommodation sharing (Guttentag et al., 2018; Lee & Kim, 2018; Li et al., 2021; Xu, 2020; Yu et al., 2020), it is not clear how the integration of innovative devices could have changed the users’ perception of the services on-offer. Staying in accommodation enhanced with advanced technologies, such as smart homes, offers value-added services due to the different degree of automation, interactivity, intelligent control and monitoring (Marikyan et al., 2019; Sequeiros et al., 2021). These functions enable users to control the consumption of water and power, reduce costs and enhance comfort (Hsu & Lin, 2016). Hence, the utilisation of the technology in peer-to-peer accommodation can add to the sustainability feature to services, result in financial benefits, improve the management of facilities, and ensure a greater degree of responsiveness to the occupants’ needs. These benefits redefine the attributes of smart accommodation and affect stay experiences, thus creating different value for their visitors.

### Smart Homes

A smart home is *“a residence equipped with a high-tech network, linking sensors and domestic devices, appliances, and features that can be remotely monitored, accessed or controlled, and provide services that respond to the needs of its inhabitants”* (Balta-Ozkan et al., 2013). The automation, control and monitoring of residents’ behaviour in smart homes bring financial, environmental, operational and psychological benefits (Balta-Ozkan et al., 2014; Marikyan et al., 2019). Financial benefits are reaped when the utilisation of smart homes helps users monitor and automate the use of electrical devices, leading to the reduction of expenses on energy consumption (Balta-Ozkan et al., 2014). The control of energy usage lowers the demand for electricity, contributing to the reduction in carbon emissions in the long term. Operational benefits are associated with the efficiency, convenience and comfort that the use of technology brings in managing daily tasks (Marikyan et al., 2019). Users experience psychological benefits, such as a positive affective state and wellbeing, when the utilisation of the technology satisfies their needs (Marikyan et al., 2020).

The benefits of smart home technologies form utilitarian and hedonic values. These have been confirmed empirically as the drivers of the use of the technology (Aldossari & Sidorova, 2020; Marikyan et al., 2021; Sequeiros et al., 2021). Specifically, it was found that intention to use smart home devices depends on hedonic value, which is the degree to which the operation of devices can induce positive feelings, such as enjoyment and pleasure (Sequeiros et al., 2021). Utilitarian value captures users’ perception of the quality and the usefulness of the product for convenience, economic savings, time efficiency and other functional purposes (Aldossari & Sidorova, 2020). It enhances the perception of the fit of technology to users’ needs, positively influencing behaviour (Marikyan et al., 2021).

Apart from value, the literature on the adoption of smart homes has examined the role of the perceived risks, behavioural beliefs and external factors determining the predisposition to use technology (Marikyan et al., 2021; Yang et al., 2017). For example, internet connectedness makes the technology vulnerable to cyber-security attacks and may compromise private data. Hence, privacy and security concerns negatively affect use behaviour (Yang et al., 2017). Behavioural beliefs underpinning motivation refer to the perception of the degree to which technology is helpful in implementing tasks, and how easy and enjoyable it is to use (Aldossari & Sidorova, 2020; Yang & Lee, 2019). External factors include social influence, underpinning intention to use technologies, which refers to the perception that the use of such technologies is deemed important by most people (Aldossari & Sidorova, 2020; Yang et al., 2017).

Given the above, the intention to stay in smart accommodation should be examined by exploring the value that the integration of innovative technologies creates. The following section discusses our conceptual model for studying value when it comes to smart accommodation. A number of hypotheses are put forward and tested empirically.

## Conceptual Model and Hypothesis Development

### Conceptual Model

**Fig. 1:**
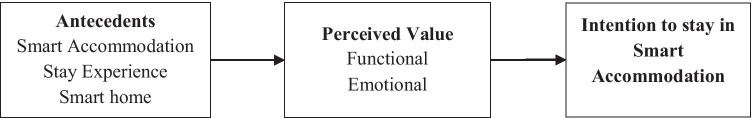
Conceptual model

This research adopts the Theory of Consumption Values, which is a theoretical framework developed by Sheth et al. (1991) that can help theoretically frame consumption behaviour in different domains (Talwar et al., 2020; Vakulenko et al., 2018; Zhang, Gu, & Jahromi, 2019b). There are three propositions of the theory guiding the conceptualisation of the research model. First, the theory postulates that individuals’ choice is affected by a combination of different consumption values: functional, social, emotional, epistemic and conditional. Functional value is the perceived utility based on the functional, utilitarian and physical characteristics of a product or a service. Social value is the perceived utility acquired from the symbolic or social associations triggered by consumption. Emotional value relates to the importance of the feelings associated with the service. Epistemic value is the utility acquired from the characteristics and the attributes of the service to create a novel experience. Conditional value represents a utility gained from a specific situation that a consumer faces (Sheth et al., 1991). Second, a consumption choice is the result of the differential effect of the above types of value (Sheth et al., 1991). This means that behaviour can be triggered by either all or some of the types of value. Given the context of this research and the empirical findings put forward by the extant literature, it is expected that staying in smart accommodation will hold emotional and functional value. The dichotomous conceptualisation of value is consistent with a research stream in the marketing field (Lee et al., 2011; Mohammad et al., 2020). Such a perspective on value captures intrinsic and extrinsic motivations, conducive to selecting to stay in smart accommodation. Third, consumption value is the reflection of the combined utility of different attributes/elements of the product or services (Sheth et al., 1991). Consequently, in line with the literature on peer-to-peer accommodation and smart homes, there are three sets of factors which could define the perception of the emotional and functional utility of smart accommodation – i.e. smart accommodation, stay-related factors and smart home factors (Aldossari & Sidorova, 2020; Böcker & Meelen, 2017; Guttentag et al., 2018; Lee & Kim, 2018; Li et al., 2021; Marikyan et al., 2021; Wang & Nicolau, 2017; Xu, 2020; Yu et al., 2020). Smart accommodation factors refer to the different characteristics and benefits of smart accommodation compared to non-smart ones. Stay factors aim to capture the experiences enabled by the integration of smart home technologies. Finally, smart home factors embrace the beliefs about the use of devices. The combinations of the factors have distinct effects on perceived functional and emotional value, contributing to intention (Fig. 1). The conceptual model is operationalised in a number of hypotheses in the sections following.

### The Antecedents of Perceived Functional Value

The integration of smart home technologies changes the functional attributes and features of accommodation, potentially affecting the perception of price value, service quality and sustainability. Price value represents the evaluation of the trade-off between the cost of the service or product and its benefits. High evaluation of benefits against the price paid for the offering positively correlates with consumption behaviour (Zeithaml, 1988). The benefits may refer to the monetary savings that an individual can gain by purchasing and using goods (Gupta et al., 2018). Also, benefits may capture other non-economic utility that products and services offer to their user (Hsu & Lin, 2016). Price value has been confirmed as an important predictor of intention to use information systems (Aldossari & Sidorova, 2020). Price has been found to significantly correlate with the motivation to adopt technologies, such as smart homes, smart applications and smartphones (Baishya & Samalia, 2020; Gupta et al., 2018; Sequeiros et al., 2021). Perceived service quality concerns the customers’ assessment of the quality of an information system or the interaction with a vendor (Dabholkar, 1996). Service quality is an important concept in both the marketing and the information systems domains (Li & Shang, 2020; Lin et al., 2017; Xu et al., 2013). When service performance falls short of expectations on any quality dimension, such as responsiveness to customers’ requirements and the reliability of services, individuals have a low likelihood of using the service again (Parasuraman et al., 1994; Xu et al., 2013). When it comes to the use of technology, service quality is positively associated with the perception of its usefulness, contributing to use intention and positive attitude (Xu et al., 2013). Perceived sustainability refers to the perception of the degree to which the performance of a service or product is environmentally friendly (Chen, Sun, et al., 2020b). The sustainability motive is one of the determinants of users’ participation in the sharing economy (Hamari et al., 2016), although this concept has not been researched explicitly in the context of peer-to-peer accommodation. The deployment of smart home technologies in sharing-based accommodation can enhance the perception of service quality, price value and performance sustainability, due to the capability of technology to reduce energy consumption, improve comfort and operational convenience (Hsu & Lin, 2016). Given the functional nature of such benefits, it is assumed that they will enhance the perceived functional value of accommodation. Therefore, the first hypothesis states that:

- H1: a) Perceived price value, b) service quality and c) sustainability are positively related to the perceived functional value of smart accommodation.

From the operational perspective, the use of smart devices in accommodation offered by sharing economy platforms can enhance users’ control of stay experiences. This assumption derives from the inherent degree of personalisation of smart home services, enabled by remote and voice control, automation and intelligent monitoring (Balta-Ozkan et al., 2013; Marikyan et al., 2019). For example, voice assistants can implement a custom-tailored service as per the command of users (Manoharan & Natu, 2021). By connecting voice-enabled assistants with other smart devices, guests can control the implementation of other services, such as light and music management, kitchen appliances and heating systems. Hence, the greater degree of the customisation of services provides a higher level of control over experience in smart accommodation compared to a traditional setting. On the other hand, the embeddedness of smart sensors in a private environment entails concerns related to potential surveillance and, in turn, the third-party use of personal data (Asaithambi et al., 2021). Perceived surveillance can reduce the functional value of smart accommodation. The perception of being watched raises uncertainty about the way that any personal information collected will be used (Jung et al., 2021). Such a concern could decrease the willingness to engage with smart devices. Given the above, this paper proposes that:

- H2: a) Perceived control over experience is positively related and b) perceived surveillance is negatively related to the perceived functional value of smart accommodation.

The smart home factors underpinning the functional value of smart accommodation include perceived external control, perceived usefulness and perceived ease of use. These beliefs concern the instrumentality of the technology in goal achievement (Davis, 1989; Venkatesh, 2000). Specifically, perceived external control refers to the situational enablers, such as resources and opportunities, which can facilitate behaviour (Venkatesh, 2000). When it comes to the use of smart home technologies, such opportunities may include manually adjustable functions that provide certainty in achieving an expected outcome. The belief that such opportunities exist is an important driver of use intention (Sintov & Schultz, 2017). Perceived usefulness is the belief that technology will help users to work more quickly, improve the performance in tasks, increase productivity and make the task easier. Perceived ease of use is the belief that the utilisation of technology is easy to learn, controllable and understandable, and the user has the required skills to operate it without effort (Davis, 1989). Perceived usefulness and perceived ease of use are the core of technology acceptance research due to their strength in explaining human interaction with information systems (Hubert et al., 2019; Shin et al., 2018). The research on smart homes has confirmed that the beliefs about technology usefulness and ease of operation form positive attitudes (Shin et al., 2018; Shuhaiber & Mashal, 2019), perceived value (Hsu & Lin, 2018), satisfaction, intention (Gu et al., 2019; Nikou, 2019) and use behaviour (Marikyan et al., 2021). Given the above evidence, the third hypothesis states that:

- H3: a) Perceived external control, b) perceived usefulness and c) perceived ease of use are positively related to the perceived functional value of smart accommodation.

### The Antecedents of Perceived Emotional Value

The sustainability benefits that smart accommodation could potentially offer have not only functional, but also emotional value. The relationship between perceived sustainability and emotional value can be explained in two ways (Ahn et al., 2020; Steg et al., 2014). First, given the energy-efficiency of smart homes, accommodation with smart devices installed can act as a cue signalling the pro-environmental benefits of its exploitation. Pro-environmental behaviour can be enjoyable per se (Steg et al., 2014). Enjoyment can be associated with the satisfaction with one’s own behaviour, stemming from the belief that the actions of an individual will result in better outcomes (Ahn et al., 2020; Lindenberg, 2001). Second, pro-environmental behaviour can be considered worthwhile because it is consistent with social norms. Acting in line with the expectations of society can make the use of environmentally sustainable products and services pleasant and enjoyable (Young, 2000). Hence, the following hypothesis states that:

- H4: Perceived sustainability has a positive relationship with the perceived emotional value of smart accommodation.

Perceived control over experience, entertainment experience, perceived surveillance and aesthetics refer to the factors contributing to the emotional value of smart accommodation. They reflect the aspects of experience in sharing-based accommodation produced during guests’ interaction with smart home technologies. Specifically, perceived control over experience is an aspect of value co-creation in sharing-based accommodation (Zhang et al., 2020). This concept reflects the participative role of an individual in creating an experience to satisfy one’s own needs (Faranda, 2001; Zhang et al., 2020). The sense of control is associated with the feeling of empowerment and predicts satisfaction (Liu & Shrum, 2002; Zhang et al., 2020). Given the higher degree of user control provided by smart devices, it is assumed that in smart accommodation guests will have an important role in co-creating their stay experience, and, in turn, emotional value. The effect of entertainment experience and aesthetics on emotional value is in line with the study by Pine et al. (1999), who differentiated the hedonic qualities of tangible and intangible offerings. In smart accommodation, entertainment reflects an individual’s perception that the use of smart devices will improve the accommodation experience. Aesthetics reflects the perception that smart devices (e.g. the way that smart lighting can create a very varied setting depending on user preference compared to conventional lighting) will be appealing to the senses. These experience dimensions have been extensively examined in the tourism and hospitality literature (Chang, 2018; Hwang & Lee, 2019; tom Dieck et al., 2018). It was confirmed that entertainment and aesthetics are important in creating a memorable experience and customer satisfaction, which, in turn, contribute to stronger engagement with the service/product (tom Dieck et al., 2018). The relationship between perceived surveillance and emotional value is expected to be negative. The risk of being the subject of surveillance induces discomfort and stress, which negatively affect satisfaction (Jung et al., 2021). Negative emotions potentially diminish the emotional value of smart accommodation. Considering the above findings, the study hypothesises that:

- H5: a) Perceived control over experience, b) entertainment experience and c) aesthetics are positively related to the emotional value of smart accommodation, while d) perceived surveillance is negatively related to it.

Perceived playfulness is the main construct explaining the intrinsic motivation of the interaction with technology (Venkatesh, 2000). It captures the aspect of the system utilisation relating to the feeling of pleasure and satisfaction. This concept measures the degree to which the use of technology is spontaneous (Webster & Martocchio, 1992). The playfulness of human-system interaction is system-independent. It is contingent on individuals’ efforts to make engagement with technology playful (Venkatesh, 2000), and on the degree of service interactivity offered by technology (Kang et al., 2020). Numerous findings provide evidence about the importance of perceived playfulness in technology acceptance (Han et al., 2020; Kang et al., 2020). It can directly affect intention to use a system (Han et al., 2020) and indirectly motivate behaviour by increasing its utility (Lin & Yeh, 2019). In line with the above evidence, it is expected that the integration of smart home technologies in accommodation will introduce novel ways of using its facilities. Due to the high degree of interactivity with the environment embedded in smart devices (Hsu & Lin, 2016; Sequeiros et al., 2021), it is more likely that individuals will perceive the engagement with the technology as playful and emotionally valuable. Given the above, we hypothesise that:

- H6: Perceived playfulness is positively related to the perceived emotional value of smart accommodation.

### Intention to Stay in Smart Accommodation

In line with the Theory of Consumption Values, the intention to stay in smart accommodation is defined by the extent to which the choice represents functional and emotional value. The role of these types of value in triggering the interaction with technology has been confirmed by multiple pieces of evidence in the information system management research (Han et al., 2017; Kim et al., 2011). In this study, perceived functional value derives from the associated operational, physical and utilitarian benefits of smart homes. Operational benefits are rooted in the automation and remote control offered by smart home devices (Marikyan et al., 2019). The deployment of such devices in sharing-based accommodation can help users enjoy the efficiency of practices inside the accommodation. The assistance and support provided by technology relieve people from the physical burden of the manual implementation of tasks (Amiribesheli et al., 2015). These services can help organise the stay in accommodation in such a way as to receive a better experience. Perceived emotional value reflects the belief that staying in smart accommodation will result in positive emotions associated with the interaction with smart home technologies. Given the above and the findings of prior research confirming the positive correlation of the hedonic and utilitarian value of smart homes on intention (Aldossari & Sidorova, 2020; Marikyan et al., 2021; Sequeiros et al., 2021), this study hypothesises that:

- H7: a) Perceived functional value and b) emotional value of smart accommodation are positively related to the intention to stay in smart accommodation.

**Fig. 2:**
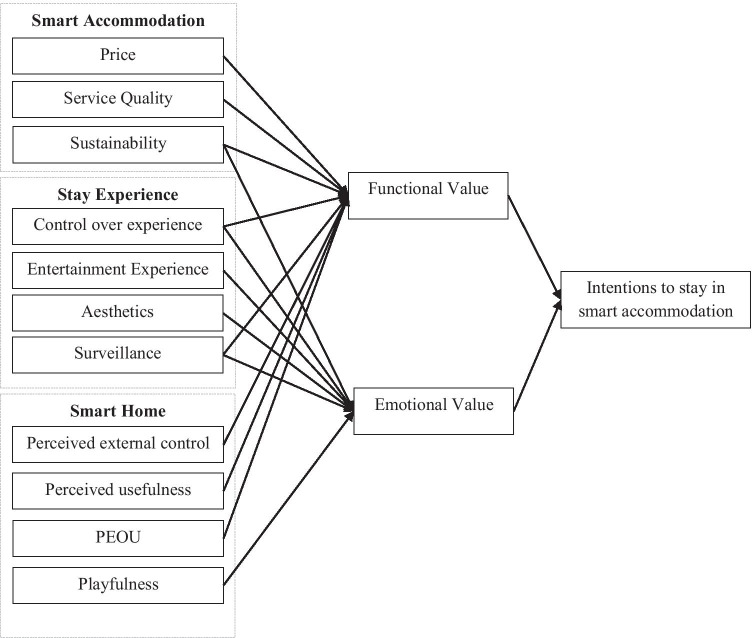
Research model

Figure 2 presents the research model with the hypothesised relationships between the antecedents of functional and emotional values underpinning intention to stay in smart accommodation.

## Methodology

### Data Collection and Sampling

A survey was used for collecting data from a consumer panel of smart homes users. The focus on smart home users ensured that respondents had had prior first-hand experience and knowledge of the technology. The questionnaire consisted of two parts. The first part contained the measures of the main concepts of the model. The second part included the questions about the socio-demographic characteristics of the respondents, the usage of smart home devices and the experience of staying in smart accommodation. 430 valid responses were collected. Table 1 presents the profile of the respondents.

**Table 1:** Demographic profile of respondents

Demographic characteristic	Type	Frequency (*n* = 430)	Percentage
Gender	Male	287	66.7
	Female	141	32.8
	Other	2	0.5
Age	under 20	70	16.3
	20–29	217	50.5
	30–39	101	23.5
	40–49	26	6.0
	50–59	11	2.6
	Over 60	5	1.2
Education	Completed some high school	17	4.0
	High school graduate or equivalent	108	25.1
	Completed some college (GSCE/AS/A-Level)	75	17.4
	Bachelor’s degree	139	32.3
	Master’s degree	80	18.6
	Other advanced degree beyond a Master’s degree	3	0.7
	Ph.D.	8	1.9
Household Income	£0 - £24,999	219	50.9
	£25,000 - £49,999	123	28.6
	£50,000 - £74,999	52	12.1
	£75,000 - £99,999	18	4.2
	More than £100,000	18	4.2
Years of use of smart home technologies	1 year ago	65	15.1
	2 years ago	110	25.6
	3 years ago	90	20.9
	4 years ago	63	14.7
	5 years ago	53	12.3
	6 years ago	9	2.1
	More than 6 years ago	40	9.3
The length of stay in accommodation (days)	1–10	286	66.5
	11–20	92	21.4
	21–30	30	7.0
	More than 30	22	5.1
Prior experience of staying in smart accommodation	Yes	241	56.0
	No	189	44.0
The usage of smart home devices while staying in smart accommodation	Smart speakers	213	88.4
	Smart camera	137	56.8
	Smart lighting	226	93.8
	Smart thermostat	223	92.5
	Smart door locks	198	82.2
	Smart pet care	72	29.9
	Smart plugs, sockets, switches and routers	187	77.6
	Smart voice-controlled assistants	188	78.0
	Smart kitchen and home appliances	182	75.5
	Smart fitness devices	131	54.4
	Smart alarms	158	65.6
	Smart bed	119	49.4
	Smart air control systems	180	74.7
	Grocery ordering (e.g. Amazon dash buttons)	99	41.1
	Smart water sprinkler, irrigation controller	96	39.8
Price willing to pay for smart accommodation	Less	34	7.9
	Same as for peer-to-peer accommodation not featuring smart home technologies	112	26.0
	More	284	66.0
Smart home technology preferences in smart accommodation	Smart speakers	343	79.8
	Smart camera	169	39.3
	Smart lighting	364	84.7
	Smart thermostat	336	78.1
	Smart door locks	311	72.3
	Smart pet care	98	22.8
	Smart plugs, sockets, switches and routers	294	68.4
	Smart voice-controlled assistants	291	67.7
	Smart kitchen and home appliances	309	71.9
	Smart fitness devices	207	48.1
	Smart alarms	258	60.0
	Smart bed	245	57.0
	Smart air control systems	300	69.8
	Grocery ordering (e.g. Amazon dash buttons)	143	33.3
	Smart water sprinkler, irrigation controller	148	34.4
Travel purpose	Leisure	309	95.1
	Business	199	46.3

### Measurements

Table 2 presents the items of the scales that were used to measure 14 constructs. All items were measured using a Likert scale with anchors between “1 – strongly disagree” to “7 – strongly agree”.

**Table 2:** Measurement items

Measurement item	Loa-ding	C.R.	AVE	Cron-bach’s α
Price Value (Sweeney & Soutar, 2001)		0.768	0.526	0.76
*Smart accommodation offered* via *sharing economy platforms ….* *will be reasonably priced*	0.655			
*will offer value for money*	0.79			
*will offer a good service for the price*	0.725			
Perceived sustainability (Chen, Sun, et al., 2020b; Hamari et al., 2016)		0.908	0.665	0.907
*Smart accommodation offered* via *sharing economy platforms ….will be friendly to the environment and harmless for nature*	0.827			
*will be environmentally friendly in terms of energy saving*	0.811			
*will be “green” and harmless for the environment*	0.838			
*will make the use of accommodation facilities more sustainable*	0.769			
*will be more ecological in terms of the use of accommodation facilities*	0.831			
Service Quality (Brady et al., 2002)		0.888	0.614	0.884
*The integration of smart home technologies will make the services of smart accommodation offered* via *sharing economy platforms …* *excellent*	0.728			
*superior, compared to traditional accommodation services offered* via *sharing economy platforms*	0.698			
*high standard*	0.838			
*high quality*	0.862			
*one of the best in the area of travel accommodation services*	0.78			
Control over experience (Zhang et al., 2020)		0.908	0.622	0.907
*Staying in smart accommodation offered* via *sharing economy platforms will make me feel that …* *I am in control of my stay experience*	0.78			
*I am in charge of my stay experience*	0.793			
*the decisions involved in my stay experience are in my hands*	0.818			
*I have control over the decisions involved in my stay experience*	0.83			
*things related to my stay experience are under my control*	0.806			
*I have influence over the things that can affect my stay experience*	0.698			
Entertainment Experience (Oh et al., 2007)		0.914	0.727	0.908
*Staying in smart accommodation offered* via *sharing economy platforms …* *will be fun*	0.882			
*will be entertaining*	0.928			
*will be enjoyable*	0.859			
*will be amusing*	0.729			
Surveillance (Jung et al., 2021)		0.958	0.821	0.957
*Staying in smart accommodation offered* via *sharing economy platforms …* *will make me feel concerned that I am being a subject of surveillance*	0.848			
*will make me feel concerned that I am being observed*	0.952			
*will make me feel concerned that I am being exposed to monitoring*	0.951			
*will make me feel concerned that my behaviour is being watched*	0.934			
*will make me feel concerned that smart devices are collecting information about me*	0.839			
Aesthetics (Oh et al., 2007)		0.876	0.702	0.876
*Staying in smart accommodation offered* via *sharing economy platforms …* *will give me pleasure while there*	0.837			
*will make my stay an attractive one*	0.855			
*will be pleasant to my senses*	0.822			
Perceived external control (Venkatesh, 2000)		0.840	0.567	0.839
*When staying in smart accommodation offered* via *sharing economy platforms …* *I will have the resources necessary to use smart home technologies*	0.765			
*I will have the knowledge necessary to use smart home technologies*	0.743			
*given the resources, opportunities and knowledge, it will be easy for me to use smart home technologies.*	0.76			
*smart home technologies will be compatible with other devices I may use while there*	0.744			
Perceived playfulness (Venkatesh & Bala, 2008)		0.860	0.609	0.854
*The usage of smart home technologies in accommodation offered* via *sharing economy platforms …* *will be spontaneous*	0.647			
*will be creative*	0.901			
*will be playful*	0.825			
*will be original*	0.725			
Perceived usefulness (Venkatesh & Bala, 2008)		0.879	0.644	0.879
*The usage of smart home technologies in accommodation offered* via *sharing economy platforms …* *will improve the performance of the tasks relevant to my stay*	0.792			
*will increase my productivity when I engage in tasks relevant to my stay*	0.814			
*will make me accomplish tasks relevant to my stay more quickly than in traditional accommodation offered* via *sharing economy platforms*	0.765			
*will be useful for accomplishing tasks relevant to my stay*	0.838			
Perceived ease of use (Venkatesh & Bala, 2008)		0.861	0.609	0.857
*The usage of smart home technologies in accommodation offered* via *sharing economy platforms …* *will be clear and understandable*	0.779			
*will not require a lot of my mental effort*	0.678			
*will be easy to use*	0.826			
*It will be easy to get smart home technologies to do what I want them to do.*	0.83			
Emotional Value (Sanchez et al., 2006; Sweeney & Soutar, 2001)		0.924	0.669	0.922
*The integration of smart home technologies in accommodation offered* via *sharing economy platforms …* *will make me comfortable with my stay experience*	0.814			
*will satisfy my wishes in relation to what I want from my stay experience*	0.824			
*will give me positive feelings*	0.837			
*will make me feel relaxed*	0.848			
*will not entail any pressure involved in organising my stay*	0.717			
*will make me feel good*	0.86			
Functional Value (Zhang et al., 2018)		0.869	0.689	0.868
*Staying in smart accommodation offered* via *sharing economy platforms …* *is convenient for my stay experience*	0.765			
*provides up-to-date facilities to satisfy my stay needs*	0.825			
*fits my stay needs*	0.895			
Intention (Venkatesh & Bala, 2008)		0.931	0.819	0.93
*I intend to stay in smart accommodation offered* via *sharing economy platforms*	0.89			
*I predict that I will book to stay in smart accommodation offered* via *sharing economy platforms*	0.939			
*I plan to book smart accommodation offered* via *sharing economy platforms*	0.885			

## Results and Analysis

### Data Analysis

To ensure the reliability and validity of the data, CFA fit indices, validity and reliability coefficients were obtained, and the possibility of common method bias was tested. The CFA model fit indices, such as Chi-Square test results, incremental fit index and absolute fit index, were satisfactory: χ2(1571) = 2935.04, P = .000, CMIN/DF = 1.880, CFI = 0.929, RMSEA = 0.045. The validity and reliability of the measurements were confirmed by factor loadings (> 0.6), construct reliability (between 0.768 and 0.958), and Cronbach’s α (> 0.76), which were above the acceptable threshold (Hair, 2014). Average variance extracted coefficients were higher than the cut-off point (AVE > 0.5). Cronbach’s α, CR and AVE values are shown in Table 2. The results of convergent and discriminant validity are presented in Table 3, where diagonal bold figures show that variance-extracted estimates are greater than the squared correlation estimates between the constructs. We have also checked for common method bias. The results of Harman’s single-factor test showed that the total variance extracted by one factor was 37%, which is less than the acceptable threshold of 50%.

**Table 3:** Convergent and discriminant validity test

	1	2	3	4	5	6	7	8	9	10	11	12	13	14
1.Functional Value	**0.830**													
2.Control over experience	0.584	**0.789**												
3.Price Value	0.522	0.422	**0.725**											
4.Perceived Sustainability	0.356	0.340	0.416	**0.816**										
5.Service Quality	0.624	0.603	0.588	0.406	**0.784**									
6.Entertainment	0.533	0.485	0.431	0.298	0.635	**0.853**								
7.Survaillance	−0.176	−0.151	−0.093	−0.089	−0.085	−0.093	**0.906**							
8.Aesthetics	0.688	0.568	0.488	0.318	0.688	0.667	−0.143	**0.838**						
9.Perceived External Control	0.607	0.538	0.453	0.329	0.556	0.534	−0.235	0.520	**0.753**					
10.Perceived Playfulness	0.522	0.405	0.393	0.233	0.497	0.620	−0.127	0.656	0.435	**0.780**				
11.Perceived Usefulness	0.676	0.571	0.454	0.325	0.628	0.544	−0.156	0.638	0.564	0.542	**0.803**			
12.PEOU	0.596	0.518	0.512	0.332	0.589	0.477	−0.224	0.544	0.743	0.426	0.600	**0.781**		
13.Emotional Value	0.766	0.641	0.538	0.376	0.712	0.656	−0.268	0.791	0.665	0.669	0.765	0.691	**0.818**	
14.Behavioural Intention	0.592	0.412	0.420	0.286	0.420	0.407	−0.111	0.461	0.455	0.384	0.505	0.453	0.593	**0.905**

Notes: Diagonal figures represent the square root of the average variance extracted (AVE) and the figures below represent the between-constructs correlations

### Path Analysis

**Fig. 3:**
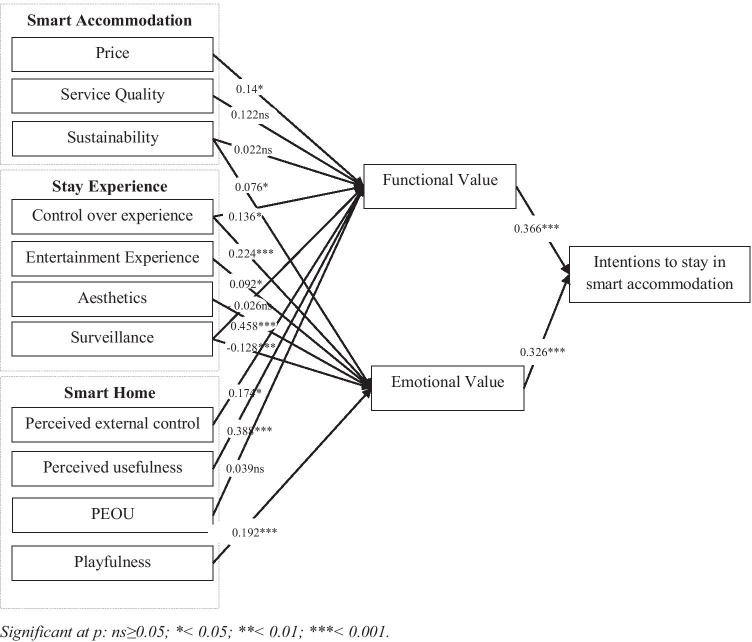
Path analysis results. Significant at p: ns ≥ 0.05; * < 0.05; ** < 0.01; *** < 0.001

The analysis of the structural model showed that model fit indices were satisfactory: χ2(1581) = 3064.9, P = .000, CMIN/DF = 1.939, CFI = 0.924, RMSEA = 0.047. The model explained 86% of the variance for Intention to Stay in smart accommodation, 17% of the variance for Perceived Emotional Value and 26% for Perceived Functional Value. Out of 16 paths, 12 were statistically significant. The path analysis results are summarised in Table 4 and illustrated in Fig. 3.

**Table 4:** Path analysis results

Hypotheses	Path			Coef.	(t-test)
H1a	Price Value	-->	Functional value	0.14	(2.371*)
H1b	Service Quality	-->	Functional value	0.122	(1.874 ns)
H1c	Perceived sustainability	-->	Functional value	0.022	(0.502 ns)
H2a	Control over experience	-->	Functional value	0.136	(2.474 *)
H2b	Surveillance	-->	Functional value	−0.026	(−0.672 ns)
H3a	Perceived external control	-->	Functional value	0.174	(2.409 *)
H3b	Perceived usefulness	-->	Functional value	0.338	(5.472 ***)
H3c	Perceived ease of use	-->	Functional value	0.039	(0.529 ns)
H4	Perceived sustainability	-->	Emotional value	0.076	(2.307 *)
H5a	Control over experience	-->	Emotional value	0.224	(5.498 ***)
H5b	Entertainment	-->	Emotional value	0.092	(1.994 *)
H5c	Aesthetics	-->	Emotional value	0.458	(7.811 ***)
H5d	Surveillance	-->	Emotional value	−0.128	(−4.285 ***)
H6	Perceived playfulness	-->	Emotional value	0.192	(4.036 ***)
H7a	Functional value	-->	Behavioural intention	0.366	(6.24 ***)
H7b	Emotional value	-->	Behavioural intention	0.326	(5.768 ***)

Significant at *p*: ns ≥ 0.05; * < 0.05; ** < 0.01; *** < 0.001

## Discussion

### The Antecedents of Perceived Functional Value

The functional value of smart accommodation is underpinned by perceived price value, control over experience, external control and perceived usefulness, representing the characteristics of accommodation, stay experience and technology utilisation. The belief that smart accommodation provided through sharing economy platforms represents high value for the price is important for potential renters because they expect the stay to bring better utilitarian benefits. Similar findings were provided by prior research establishing a relationship between price value, tourist consumption and technology use choices (Aldossari & Sidorova, 2020; Choe & Kim, 2018). The study demonstrates that renters are willing to pay a higher price for accommodation featuring smart home devices. This insight is especially important for the current debate about the hosts’ benefits and risks of embracing smart home technology (Pappas et al., 2021; Pizam, 2017), as it provides a customer’s perception of the utility of such technology and serves as a rationale for its adoption in the accommodation sector. Perceived service quality and sustainability are not important attributes for building the functional value of smart accommodation. The insignificant role of perceived service quality is inconsistent with the findings that the assessment of the quality of product and service predefines attitude and use intention (Li & Shang, 2020; Lin et al., 2017; Xu et al., 2013). While the smart accommodation services may provide some relative advantages in terms of price and experience, the overall service is not evaluated as superior to traditional sharing-based accommodation. The insignificant path between perceived sustainability and functional value contradicts the research concluding that the expected sustainability of smart homes predicts its adoption (Hsu & Lin, 2016). The installation of technology potentially leading to energy preservation and carbon footprint reduction (Hsu & Lin, 2016) does not create a functional value for smart accommodation. Accommodation featuring smart devices is not perceived as a sustainable service, although traditional peer-to-peer accommodation is associated with a sustainable lifestyle, triggering customers’ interest (Serrano et al., 2021). That means that individuals’ perceptions of the technology are defined by the purpose for which technology is employed, thus providing new evidence for the literature on the applications of smart homes (Aldossari & Sidorova, 2020; Hussain et al., 2009; Marikyan et al., 2021). In particular, this result can be explained in two ways. First, sustainability implications require not only the use of energy-efficient technology, but mindful consumption behaviour (Vlachokostas, 2020). Respondents may believe that short-stays do not have much of an environmental impact in their own right. Secondly, compared to the home settings, people might have different consumption patterns in the accommodation context. Given that sustainability is the long-term implication of cost-reduction measures using smart homes (Marikyan et al., 2019), it is very likely that in smart accommodation people are less concerned about monetary spending. Guests might be less motivated to monitor the use of resources because they often pay for energy consumption upfront as an added fixed cost included in the accommodation price.

The established role of perceived control over experience means that the capability of smart homes to offer better service personalisation, control, automation and monitoring (Balta-Ozkan et al., 2013; Marikyan et al., 2019) enhances the belief that guests will have the opportunity to customise their stay experience to their requirements. This evidence enriches the literature on the role of innovative technology in optimising and improving tourist experiences (Buhalis & Leung, 2018; Jeong & Shin, 2020; Leung, 2019; Pai et al., 2020). For example, it was found that smart mobile technologies tailor experiences by navigating tourists in travel destinations (Jeong & Shin, 2020). Smart home devices and sensors have implications for hotel management, who use real time data collected by technology for understanding the needs of customers and personalising services (Buhalis & Leung, 2018). This study, in turn, confirms the value that smart technology functions offer for customising renters’ experiences inside accommodation through enhanced control. The insignificant correlation of perceived surveillance with functional value suggests that for the majority of renters, potential surveillance using smart devices (i.e. smart cameras) does not diminish the utility of technology during the stay. This finding contradicts the literature discussing the risk of privacy intrusion and uncertainty concerns when using smart home systems (Asaithambi et al., 2021; Jung et al., 2021). Considering the preferences of respondents, scoring smart cameras as one of the least preferred devices, it is likely that renters would not feel comfortable using cameras in accommodation. However, they would still perceive the functional benefits of this device integration. More data with regard to the ability to turn cameras on or off and their positioning (e.g. internal or external) may have helped put such a finding into context.

According to the analysis of smart home factors, the existence of external control and the perception of technology usefulness are important to ensure the functional utility of smart accommodation. As highlighted by prior research (Sintov & Schultz, 2017; Venkatesh, 2000), guests need to know that they have the resources and opportunities (e.g. manual control, guide) to ensure the delivery of services. The confirmed path between perceived usefulness and functional value means that smart home technologies are instrumental for implementing the tasks inside accommodation that are relevant to their stay. The supported relationship shows that as in personal home settings (Shin et al., 2018), perceived usefulness shows a significant predictive strength when it comes to the use of technology in short-stay rental apartments. However, the insignificant role of perceived ease of use is inconsistent with evidence about the role of this factor in technology acceptance (Hsu & Lin, 2018; Shin et al., 2018; Shuhaiber & Mashal, 2019). There is a plausible explanation for such a result. Given that the responses were collected from smart home users, the complexity of technology is, most probably, not important for individuals with prior use experience and knowledge. These findings widen knowledge about users’ perception of the operational and usability aspects of smart home technology when applied in different contexts.

### The Antecedents of Perceived Emotional Value

The emotional value of smart accommodation was found to relate to five factors referring to smart accommodation attributes, stay experiences and technology use. Specifically, the sustainability feature of smart accommodation helps create an emotional value of services, which is consistent with the discussion about the motivations of pro-environmental behaviour (Ahn et al., 2020; Steg et al., 2014). This finding is in line with evidence reported in the study analysing customer reviews about hotel sustainability indicators (Saura et al., 2018). The lack of facilities and policies ensuring energy efficiency is associated with negative sentiments (Saura et al., 2018). In a similar vein, the possibility of using energy-efficient smart home devices in peer-to-peer accommodation induces positive emotions, even though it may not result in the actual implications of sustainable behaviour, as can be inferred by the insignificant correlation of perceived sustainability and functional value.

The analysis of stay factors confirms that control over experience, entertainment experience and aesthetics are expected to create positive emotions during the stay in smart accommodation. The interaction with smart home technologies increases the participatory role of guests in controlling and co-creating their stay experiences. Since individuals’ role in value-creation is associated with feelings of empowerment and satisfaction (Faranda, 2001; Liu & Shrum, 2002; Zhang et al., 2020), the perception of enhanced control contributes to positive emotions. Also, two experience dimensions were found important for potential renters, as postulated by prior research (Chang, 2018; Hwang & Lee, 2019; tom Dieck et al., 2018). People are likely to feel good about choosing smart accommodation because it is perceived to be more entertaining and aesthetically different from apartments not featuring smart home technologies. However, the perceived emotional value was found to be undermined by the respondents’ concern about surveillance through sensors and cameras. This is not surprising, since the intentional or unintentional generation of video content about customers’ behaviour by innovative technologies has been widely perceived to be a practice against ethical principles (Saura et al., 2021a; Saura et al., 2021b). Privacy concerns trigger negative emotions, such as stress and discomfort (Jung et al., 2021). Although the risk of being observed is psychologically unpleasant, it does not reduce the perception of the functional utility of smart home technologies installed in accommodation.

Finally, the correlation of perceived playfulness and emotional value shows that the use of smart home technologies in accommodation brings fun and enjoyment. This finding reflects the major discussion in information systems research about the importance of computer playfulness in making decisions regarding the use of technology (Han et al., 2020; Kang et al., 2020; Venkatesh, 2000). The interactive nature of smart homes and connectivity with other devices (Hsu & Lin, 2016; Sequeiros et al., 2021) provides guests with the opportunity to use facilities in creative and personalised ways. This, in turn, is expected to satisfy guests’ anticipation about the stay experience and raise positive emotions.

### The Perceived Value of Smart Accommodation

The path analysis shows that intention to stay in smart accommodation is underpinned by the perception of both its functional and emotional value. This finding is consistent with the Theory of Consumption Values and the technology acceptance research postulating that these values are important for predicting consumption behaviour (Sheth et al., 1991) and user-technology interaction (Han et al., 2017; Kim et al., 2011). In line with the discussion in the smart home literature (Amiribesheli et al., 2015), visitors are more likely to rent smart accommodation because the implementation of any tasks will be more efficient and convenient. The interaction with smart home technologies is more likely to result in a better stay experience and invoke positive emotions. These results provide new pieces of evidence for the literature on smart tourism and innovative technology diffusion (Li et al., 2017; Marikyan et al., 2021; Wang et al., 2020). They demonstrate the potential implications of smart home applications in peer-to-peer accommodation and the factors underpinning technology adoption in the tourism sector.

The analysis of the relationships between perceived value and intention, as well as insights into the preferences of respondents, provide evidence about the differentiating factors contributing to the motivation to stay in smart accommodation. The collected data help us understand whether the integration of innovative technologies can add value to peer-to-peer accommodation services, which have been badly affected by the pandemic (Gerwe, 2021; Hossain, 2021; Kraus et al., 2020). The majority of surveyed respondents expressed a high likelihood of using smart accommodation in the future (71%) and a willingness to pay an extra price for it (66%), thus indicating promising prospects for the new service offering. When staying in smart accommodation, the respondents would prefer to use technologies that are capable of ensuring security, automation, personalisation, remote control and monitoring, as summarised in Table 1. These functions represent operational and utilitarian benefits.

## Conclusion

This study addressed two objectives, which aimed to explore the motivation to stay in smart accommodation by examining 1) the factors relating to smart accommodation, stay experience and smart homes, creating the functional and emotional value of smart accommodation and 2) the correlation of perceived value with intention to stay in smart accommodation in the future. The analysis of the antecedents of value demonstrated that functional value is determined by four factors: the perception that smart accommodation represents a good value for the price; that it offers the opportunity to control the stay experience; that the smart technologies installed are useful; and that guests have the resources to utilise them. Emotional value depends on the perception of the sustainability of smart accommodation, control over the experience, the entertainment and aesthetic qualities of the service, and the perceived playfulness of smart home technologies. Also, emotional value negatively correlates with perceived surveillance. Finally, intention to stay in smart accommodation was underpinned by the perception that the stay would bring functional utility and a positive emotional response.

### Theoretical Contributions

The findings of the research offer three important theoretical contributions. Firstly, the paper contributes to the smart tourism research stream, discussing the conditions to catalyse the adoption of innovative technologies and services in the hospitality sector (Li et al., 2017). In contrast to prior research, which focused on smart infrastructure and ecosystems in tourism destinations (Jeong & Shin, 2020; Lee et al., 2020; Wang et al., 2020) and explored the accommodation providers’ view on the adoption of smart technologies (Pappas et al., 2021), this study explored guests’ motivations to stay in smart accommodation. These findings are important in the growing stream in the literature about the implications of innovative technologies in the tourism and hospitality industry (Dickinson et al., 2017; Li et al., 2017).

Secondly, the research contributes to the literature focusing on accidental spillover effects when it comes to new applications of innovative technologies. Specifically, as far as the smart home literature is concerned, there are limited insights into the drivers of intention to use smart home technologies in contexts beyond personal home settings (Aldossari & Sidorova, 2020; Hussain et al., 2009; Marikyan et al., 2021). On the one hand, technology utilisation in the new context potentially implies different behavioural patterns and a degree of privacy control due to the involvement of a third party in the delivery of services and the different nature of use. On the other hand, the decision about the use of technology in the new context may be influenced by their adoption preferences in the initial settings. The insights into spillover effects in technology utilisation provide important evidence for understanding user behaviour, given the wider applications of innovative technologies in the private, public and industry sectors (Budd et al., 2020; Leonardi, 2020; Papadopoulos et al., 2020).

Thirdly, the paper contributes to the debate in the literature about the implications of digital technologies for transforming service propositions and delivery (Pai et al., 2020; Usak et al., 2020). Given that published papers mostly discuss the development, application and impact of new technology designed to fight the propagation of COVID-19 (Bragazzi et al., 2020; Ribeiro-Navarrete et al., 2021), there is no research that would shed light on the adoption of innovative technologies in the accommodation sector in the scenarios of post-pandemic recovery (Gerwe, 2021). Hence, in light of the impact of the COVID-19 pandemic on the sharing economy, this paper provides timely insights into the factors that could facilitate customer interest in sharing-based accommodation services. This paper considers the features and aspects of services that are important for guests and shows the potential of the new service offering in creating value.

### Practical Implications

The findings provide practical implications for facilitating the diffusion of smart home technologies. The significant relationship between functional and emotional value and intention indicates that smart home devices can provide guests with better services and experiences. This finding can inform SMEs in the accommodation sharing sector about the positive implications of the use of such technology for driving customer demand. From a smart home provider’s point of view, smart home technologies can provide guests with the opportunities to experiment with services and try new applications, potentially raising guests’ interest in adopting the technology in their own homes.

Also, the identified antecedents provide actionable insights into the features of accommodation and the characteristics of technologies that would facilitate the creation of functional and emotional value. The finding of perceived external control demonstrates the importance of providing a device usability guide for guests to have sufficient knowledge and resources to operate technologies. Given the importance of entertainment experience and playfulness, accommodation hosts could install devices enabling a higher degree of interactivity, such as voice assistants. Considering the strong effect of aesthetics on emotional value, smart devices should fit well visually into the accommodation.

Thirdly, from the marketing perspective, to increase the awareness of the benefits of smart accommodation against costs, hosts need to ensure that listings include a description of their relative advantage compared to a traditional service. It is important to highlight the sustainability implications, as this increases the emotional value of the offering. To differentiate services, the websites could have a dedicated section and specific search criteria for smart accommodation offerings. To eliminate surveillance concerns and enhance trust towards providers, rental agreements need to include a clause about the use of the data collected.

### Limitations and Future Research Suggestions

This research has some limitations. Considering the methodological choices made, there are several areas for research that future studies can build upon. Firstly, the research model needs to be tested in different geographical locations and cultures. It is more likely that the perceptions of certain features – i.e. surveillance, price value, perceived ease of use – will vary in different countries, due to the difference in socio-economic and infrastructural development. Culture may explain the variance in expectations about aesthetic and entertainment experience and perceived sustainability effects rooted in different social norms and beliefs. Secondly, the model can be tested by controlling for the role of prior stay experience. Future research could check whether the clusters of guests who have stayed once vs regularly in peer-to-peer accommodation have different perceptions of smart accommodation features, potentially affecting their motivation to rent it. Thirdly, it will be worth testing the model by examining the intention to stay in accommodation with particular types of technology. This would make it possible to explore which technology and functions create value for guests. Fourthly, researchers could test the model with a sample of non-smart home users, because the perception of the features of smart accommodation and their functional and emotional value can be different depending on the degree of familiarity with, and the knowledge of, smart home technologies. By controlling for the role of experience of using smart home devices, it would be possible to validate whether such factors as perceived ease of use and perceived surveillance are significant predictors of intention to stay in smart accommodation.

The findings of the study offer several directions for future research. Given the insignificant role of perceived surveillance in the perception of functional value, it is worth examining the technology-user interaction patterns that could negate the potential negative aspects of monitoring through smart home devices. Also, future research could explore renters’ behaviours to understand why the sustainability feature of accommodation integrated with energy-efficient devices raises positive feelings and emotions, though it is not perceived as functional. Researchers could explore the tendency and associated motives to practice energy-efficient consumption in rented accommodation. Finally, future studies could examine the perception of different dimensions of service quality (e.g. tangibility, reliability, responsiveness) from the functional perspective. Such an approach would help identify the attributes of smart accommodation services that could undermine the evaluation of the overall quality of services.

## References

[CR1] Ahn I, Kim SH, Kim M (2020). The relative importance of values, social norms, and enjoyment-based motivation in explaining pro-environmental product purchasing behavior in apparel domain. Sustainability.

[CR2] Akbar YH, Tracogna A (2018). The sharing economy and the future of the hotel industry: Transaction cost theory and platform economics. International Journal of Hospitality Management.

[CR3] Aldossari MQ, Sidorova A (2020). Consumer acceptance of internet of things (IoT): Smart home context. Journal of Computer Information Systems.

[CR4] Amiribesheli M, Benmansour A, Bouchachia A (2015). A review of smart homes in healthcare. Journal of Ambient Intelligence and Humanized Computing.

[CR5] Asaithambi SPR, Venkatraman S, Venkatraman R (2021). Big data and personalisation for non-intrusive smart home automation. Big Data and Cognitive Computing.

[CR6] Baishya K, Samalia HV (2020). Extending unified theory of acceptance and use of technology with perceived monetary value for smartphone adoption at the bottom of the pyramid. International Journal of Information Management.

[CR7] Balta-Ozkan N, Davidson R, Bicket M (2013). Social barriers to the adoption of smart homes. Energy Policy.

[CR8] Balta-Ozkan N, Amerighi O, Boteler B (2014). A comparison of consumer perceptions towards smart homes in the UK, Germany and Italy: Reflections for policy and future research. Technology Analysis & Strategic Management.

[CR9] Belk R (2014). You are what you can access: Sharing and collaborative consumption online. Journal of Business Research.

[CR10] Birinci, H., Berezina, K., & Cobanoglu, C. (2018). Comparing customer perceptions of hotel and peer-to-peer accommodation advantages and disadvantages. *International Journal of Contemporary Hospitality Management, 30*, 1190–1210.

[CR11] Böcker L, Meelen T (2017). Sharing for people, planet or profit? Analysing motivations for intended sharing economy participation. Environmental Innovation and Societal Transitions.

[CR12] Brady MK, Cronin JJ, Brand RR (2002). Performance-only measurement of service quality: A replication and extension. Journal of Business Research.

[CR13] Bragazzi NL, Dai H, Damiani G (2020). How big data and artificial intelligence can help better manage the COVID-19 pandemic. International Journal of Environmental Research and Public Health.

[CR14] Budd J, Miller BS, Manning EM (2020). Digital technologies in the public-health response to COVID-19. Nature Medicine.

[CR15] Buhalis D, Leung R (2018). Smart hospitality—Interconnectivity and interoperability towards an ecosystem. International Journal of Hospitality Management.

[CR16] Camilleri, J., & Neuhofer, B. (2017). Value co-creation and co-destruction in the Airbnb sharing economy. *International Journal of Contemporary Hospitality Management, 29*, 2322–2340.

[CR17] Chang S (2018). Experience economy in the hospitality and tourism context. Tourism Management Perspectives.

[CR18] Chen, G., Cheng, M., Edwards, D., et al. (2020a). COVID-19 pandemic exposes the vulnerability of the sharing economy: A novel accounting framework. *Journal of Sustainable Tourism*, 1–18.

[CR19] Chen X, Sun X, Yan D (2020). Perceived sustainability and customer engagement in the online shopping environment: The rational and emotional perspectives. Sustainability.

[CR20] Choe JYJ, Kim SS (2018). Effects of tourists’ local food consumption value on attitude, food destination image, and behavioral intention. International Journal of Hospitality Management.

[CR21] Dabholkar PA (1996). Consumer evaluations of new technology-based self-service options: An investigation of alternative models of service quality. International Journal of Research in Marketing.

[CR22] Davis, F. D. (1989). Perceived usefulness, perceived ease of use, and user acceptance of information technology. *MIS Quarterly*, 319–340.

[CR23] Davlembayeva D, Papagiannidis S (2021). Paradoxes of the sharing economy: A pandemic perspective. International Review of Entrepreneurship.

[CR24] Davlembayeva, D., Papagiannidis, S., & Alamanos, E. (2019). Mapping the economics, social and technological attributes of the sharing economy. *Information Technology & People, 33*, 841–872.

[CR25] Davlembayeva D, Papagiannidis S, Alamanos E (2020). Sharing economy: Studying the social and psychological factors and the outcomes of social exchange. Technological Forecasting and Social Change.

[CR26] Deloitte. (2020). Impact of the COVID-19 crisis on short- and medium-term consumer behavior. Available at: https://www2.deloitte.com/uk/en/pages/financial-advisory/articles/the-impact-of-covid-19.html. Accessed 27 Apr 2021.

[CR27] Dickinson JE, Hibbert JF, Filimonau V (2017). Implementing smartphone enabled collaborative travel: Routes to success in the tourism domain. Journal of Transport Geography.

[CR28] Faranda WT (2001). A scale to measure the cognitive control form of perceived control: Construction and preliminary assessment. Psychology & Marketing.

[CR29] Gao F, Sunyaev A (2019). Context matters: A review of the determinant factors in the decision to adopt cloud computing in healthcare. International Journal of Information Management.

[CR30] Gerwe O (2021). The Covid-19 pandemic and the accommodation sharing sector: Effects and prospects for recovery. Technological Forecasting and Social Change.

[CR31] Gu W, Bao P, Hao W (2019). Empirical examination of intention to continue to use smart home services. Sustainability.

[CR32] Gupta A, Dogra N, George B (2018). What determines tourist adoption of smartphone apps?. Journal of Hospitality and Tourism Technology.

[CR33] Guttentag D, Smith S, Potwarka L (2018). Why tourists choose Airbnb: A motivation-based segmentation study. Journal of Travel Research.

[CR34] Hair, J. F. (2014). Multivariate data analysis (7th ed). Harlow, Essex: Prentice Hall.

[CR35] Hamari J, Sjöklint M, Ukkonen A (2016). The sharing economy: Why people participate in collaborative consumption. Journal of the Association for Information Science and Technology.

[CR36] Han L, Wang S, Zhao D (2017). The intention to adopt electric vehicles: Driven by functional and non-functional values. Transportation Research Part A: Policy and Practice.

[CR37] Han S-L, An M, Han JJ (2020). Telepresence, time distortion, and consumer traits of virtual reality shopping. Journal of Business Research.

[CR38] Hayes B, Kamrowska-Zaluska D, Petrovski A (2021). State of the art in open platforms for collaborative Urban Design and sharing of resources in districts and cities. Sustainability.

[CR39] Hossain M (2021). The effect of the Covid-19 on sharing economy activities. Journal of Cleaner Production.

[CR40] Hsu C-L, Lin JC-C (2016). An empirical examination of consumer adoption of internet of things services: Network externalities and concern for information privacy perspectives. Computers in Human Behavior.

[CR41] Hsu C-L, Lin JC-C (2018). Exploring factors affecting the adoption of internet of things services. Journal of Computer Information Systems.

[CR42] Hubert, M., Blut, M., Brock, C., et al. (2019). The influence of acceptance and adoption drivers on smart home usage. *European Journal of Marketing, 53*, 1073–1098.

[CR43] Hussain S, Erdogen SZ, Park JH (2009). Monitoring user activities in smart home environments. Information Systems Frontiers.

[CR44] Hwang J, Lee J (2019). A strategy for enhancing senior tourists’ well-being perception: Focusing on the experience economy. Journal of Travel & Tourism Marketing.

[CR45] Jeong M, Shin HH (2020). Tourists’ experiences with smart tourism technology at smart destinations and their behavior intentions. Journal of Travel Research.

[CR46] Jiang Y, Balaji M, Jha S (2019). Together we tango: Value facilitation and customer participation in Airbnb. International Journal of Hospitality Management.

[CR47] Johnson, A.-G., & Neuhofer, B. (2017). Airbnb–an exploration of value co-creation experiences in Jamaica. *International Journal of Contemporary Hospitality Management, 29*, 2361–2376.

[CR48] Jung Y, Choi B, Cho W (2021). Group satisfaction with group work under surveillance: The stimulus-organism-response (SOR) perspective. Telematics and Informatics.

[CR49] Kang HJ, J-h S, Ponto K (2020). How 3D virtual reality stores can shape consumer purchase decisions: The roles of Informativeness and playfulness. Journal of Interactive Marketing.

[CR50] Kim H-W, Gupta S, Koh J (2011). Investigating the intention to purchase digital items in social networking communities: A customer value perspective. Information & Management.

[CR51] Kraus, S., Clauss, T., Breier, M., et al. (2020). The economics of COVID-19: Initial empirical evidence on how family firms in five European countries cope with the corona crisis. *International Journal of Entrepreneurial Behavior & Research, 26*, 1067–1092.

[CR52] Lee, S., & Kim, D.-Y. (2018). The effect of hedonic and utilitarian values on satisfaction and loyalty of Airbnb users. *International Journal of Contemporary Hospitality Management, 30*, 1332–1351.

[CR53] Lee J-S, Lee C-K, Choi Y (2011). Examining the role of emotional and functional values in festival evaluation. Journal of Travel Research.

[CR54] Lee P, Hunter WC, Chung N (2020). Smart tourism city: Developments and transformations. Sustainability.

[CR55] Leonardi, P. M. (2020). COVID-19 and the new technologies of organizing: Digital exhaust, digital footprints, and artificial intelligence in the wake of remote work. *Journal of Management Studies, 58*, 247–251.

[CR56] Leung, R. (2019). Smart hospitality: Taiwan hotel stakeholder perspectives. *Tourism Review, 74*, 50–62.

[CR57] Li Y, Shang H (2020). Service quality, perceived value, and citizens’ continuous-use intention regarding e-government: Empirical evidence from China. Information & Management.

[CR58] Li Y, Hu C, Huang C (2017). The concept of smart tourism in the context of tourism information services. Tourism Management.

[CR59] Li J, Hudson S, So KKF (2021). Hedonic consumption pathway vs. acquisition-transaction utility pathway: An empirical comparison of Airbnb and hotels. International Journal of Hospitality Management.

[CR60] Lin P-H, Yeh S-C (2019). How motion-control influences a VR-supported technology for mental rotation learning: From the perspectives of playfulness, gender difference and technology acceptance model. International Journal of Human Computer Interaction.

[CR61] Lin M, Wu X, Ling Q (2017). Assessing the effectiveness of empowerment on service quality: A multi-level study of Chinese tourism firms. Tourism Management.

[CR62] Lindenberg S (2001). Intrinsic motivation in a new light. Kyklos.

[CR63] Liu Y, Shrum LJ (2002). What is interactivity and is it always such a good thing? Implications of definition, person, and situation for the influence of interactivity on advertising effectiveness. Journal of Advertising.

[CR64] Manoharan S, Natu P (2021). Development of a framework for a collaborative and personalised voice assistant. Electronic Government, An International Journal.

[CR65] Marikyan D, Papagiannidis S, Alamanos E (2019). A systematic review of the smart home literature: A user perspective. Technological Forecasting and Social Change.

[CR66] Marikyan, D., Papagiannidis, S., & Alamanos, E. (2020). Cognitive dissonance in technology adoption: A study of smart home users. *Information Systems Frontiers*, 1–23. 10.1007/s10796-020-10042-3 PMC7381864 32837263

[CR67] Marikyan D, Papagiannidis S, Alamanos E (2021). “Smart home sweet smart home”: An examination of smart home acceptance. International Journal of E-Business Research (IJEBR).

[CR68] Martin CJ (2016). The sharing economy: A pathway to sustainability or a nightmarish form of neoliberal capitalism?. Ecological Economics.

[CR69] Mohammad J, Quoquab F, Thurasamy R (2020). The effect of user-generated content quality on brand engagement: The mediating role of functional and emotional values. Journal of Electronic Commerce Research.

[CR70] Nadeem W, Juntunen M, Shirazi F (2020). Consumers’ value co-creation in sharing economy: The role of social support, consumers’ ethical perceptions and relationship quality. Technological Forecasting and Social Change.

[CR71] Nikou S (2019). Factors driving the adoption of smart home technology: An empirical assessment. Telematics and Informatics.

[CR72] Obaidat MS, Nicopolitidis P (2016). Smart cities and homes: Key enabling technologies.

[CR73] OCAO. (2021). *Air travel down 60 per cent, as airline industry losses top $370 billion: ICAO*. Available at: https://news.un.org/en/story/2021/01/1082302. Accessed 27 Apr 2021.

[CR74] Oh H, Fiore AM, Jeoung M (2007). Measuring experience economy concepts: Tourism applications. Journal of Travel Research.

[CR75] ONS. (2020). *Coronavirus and the impact on output in the UK economy: June 2020* Available at: https://www.ons.gov.uk/economy/grossdomesticproductgdp/articles/coronavirusandtheimpactonoutputintheukeconomy/june2020. Accessed 27 Apr 2021.

[CR76] Pai C-K, Liu Y, Kang S (2020). The role of perceived smart tourism technology experience for tourist satisfaction, happiness and revisit intention. Sustainability.

[CR77] Papadopoulos T, Baltas KN, Balta ME (2020). The use of digital technologies by small and medium enterprises during COVID-19: Implications for theory and practice. International Journal of Information Management.

[CR78] Pappas N, Caputo A, Pellegrini MM (2021). The complexity of decision-making processes and IoT adoption in accommodation SMEs. Journal of Business Research.

[CR79] Parasuraman A, Zeithaml VA, Berry LL (1994). Reassessment of expectations as a comparison standard in measuring service quality: Implications for further research. Journal of Marketing.

[CR80] Pine BJ, Pine J, Gilmore JH (1999). The experience economy: Work is theatre & every business a stage.

[CR81] Pizam A (2017). The internet of things (IoT): The next challenge to the hospitality industry. International Journal of Hospitality Management.

[CR82] Rathore H, Mohamed A, Guizani M (2020). A survey of blockchain enabled cyber-physical systems. Sensors.

[CR83] Ribeiro-Navarrete S, Saura JR, Palacios-Marqués D (2021). Towards a new era of mass data collection: Assessing pandemic surveillance technologies to preserve user privacy. Technological Forecasting and Social Change.

[CR84] Sanchez J, Callarisa L, Rodriguez RM (2006). Perceived value of the purchase of a tourism product. Tourism Management.

[CR85] Saura JR, Reyes-Menendez A, Alvarez-Alonso C (2018). Do online comments affect environmental management? Identifying factors related to environmental management and sustainability of hotels. Sustainability.

[CR86] Saura, J. R., Ribeiro-Soriano, D., & Palacios-Marqués, D. (2021a). From user-generated data to data-driven innovation: A research agenda to understand user privacy in digital markets. *International Journal of Information Management, 60*, 102331.

[CR87] Saura, J. R., Ribeiro-Soriano, D., & Palacios-Marqués, D. (2021b). Setting privacy “by default” in social IoT: Theorizing the challenges and directions in big data research. *Big Data Research, 25*, 100245.

[CR88] Sequeiros, H., Oliveira, T., & Thomas, M. A. (2021). The impact of IoT smart home services on psychological well-being. *Information Systems Frontiers*, 1–18. 10.1007/s10796-021-10118-8

[CR89] Serrano L, Ariza-Montes A, Nader M (2021). Exploring preferences and sustainable attitudes of Airbnb green users in the review comments and ratings: A text mining approach. Journal of Sustainable Tourism.

[CR90] Sheth JN, Newman BI, Gross BL (1991). Why we buy what we buy: A theory of consumption values. Journal of Business Research.

[CR91] Shin J, Park Y, Lee D (2018). Who will be smart home users? An analysis of adoption and diffusion of smart homes. Technological Forecasting and Social Change.

[CR92] Shuhaiber A, Mashal I (2019). Understanding users’ acceptance of smart homes. Technology in Society.

[CR93] Sintov ND, Schultz P (2017). Adjustable green defaults can help make smart homes more sustainable. Sustainability.

[CR94] Steg L, Perlaviciute G, Van der Werff E (2014). The significance of hedonic values for environmentally relevant attitudes, preferences, and actions. Environment and Behavior.

[CR95] Sundararajan A (2016). The sharing economy: The end of employment and the rise of crowd-based capitalism.

[CR96] Sweeney JC, Soutar GN (2001). Consumer perceived value: The development of a multiple item scale. Journal of Retailing.

[CR97] Talwar S, Dhir A, Kaur P (2020). Why do people purchase from online travel agencies (OTAs)? A consumption values perspective. International Journal of Hospitality Management.

[CR98] tom Dieck MC, Jung TH, Rauschnabel PA (2018). Determining visitor engagement through augmented reality at science festivals: An experience economy perspective. Computers in Human Behavior.

[CR99] Tussyadiah IP, Pesonen J (2016). Drivers and barriers of peer-to-peer accommodation stay – An exploratory study with American and Finnish travellers. Current Issues in Tourism.

[CR100] Usak M, Kubiatko M, Shabbir MS (2020). Health care service delivery based on the internet of things: A systematic and comprehensive study. International Journal of Communication Systems.

[CR101] Vakulenko Y, Hellström D, Hjort K (2018). What's in the parcel locker? Exploring customer value in e-commerce last mile delivery. Journal of Business Research.

[CR102] Venkatesh V (2000). Determinants of perceived ease of use: Integrating control, intrinsic motivation, and emotion into the technology acceptance model. Information Systems Research.

[CR103] Venkatesh V, Bala H (2008). Technology acceptance model 3 and a research agenda on interventions. Decision Sciences.

[CR104] Vlachokostas C (2020). Smart buildings need smart consumers: The meet-in-the middle approach towards sustainable management of energy sources. International Journal of Sustainable Energy.

[CR105] Wang D, Nicolau JL (2017). Price determinants of sharing economy based accommodation rental: A study of listings from 33 cities on Airbnb.com. International Journal of Hospitality Management.

[CR106] Wang J, Xie C, Huang Q (2020). Smart tourism destination experiences: The mediating impact of arousal levels. Tourism Management Perspectives.

[CR107] Webster, J., & Martocchio, J. J. (1992). Microcomputer playfulness: Development of a measure with workplace implications. *MIS Quarterly, 16*, 201–226.

[CR108] Xu X (2020). How do consumers in the sharing economy value sharing? Evidence from online reviews. Decision Support Systems.

[CR109] Xu, J., Benbasat, I., & Cenfetelli, R. T. (2013). Integrating service quality with system and information quality: An empirical test in the e-service context. *MIS Quarterly, 37*, 777–794.

[CR110] Yang H, Lee H (2019). Understanding user behavior of virtual personal assistant devices. Information Systems and e-Business Management.

[CR111] Yang, H., Lee, H., & Zo, H. (2017). User acceptance of smart home services: An extension of the theory of planned behavior. *Industrial Management & Data Systems, 117*, 68–89.

[CR112] Young, D. (2000). Expanding and evaluating motives for environmentally responsible behavior-statistical data included. *Journal of Social Issues, 56*, 509–526.

[CR113] Yu, M., Cheng, M., Yu, Z., et al. (2020). Investigating Airbnb listings’ amenities relative to hotels. *Current Issues in Tourism*, 1–18. 10.1080/13683500.2020.1733497

[CR114] Zeithaml VA (1988). Consumer perceptions of price, quality, and value: A means-end model and synthesis of evidence. Journal of Marketing.

[CR115] Zhang TC, Jahromi MF, Kizildag M (2018). Value co-creation in a sharing economy: The end of price wars?. International Journal of Hospitality Management.

[CR116] Zhang T, Bufquin D, Lu C (2019). A qualitative investigation of microentrepreneurship in the sharing economy. International Journal of Hospitality Management.

[CR117] Zhang TC, Gu H, Jahromi MF (2019). What makes the sharing economy successful? An empirical examination of competitive customer value propositions. Computers in Human Behavior.

[CR118] Zhang, P., Meng, F., & So, K. K. F. (2020). Cocreation experience in peer-to-peer accommodations: Conceptualization and scale development. *Journal of Travel Research, 60*, 1333–1351.

